# Supplementary material for: Transcriptome Analysis Reveals Key Seed-Development Genes in Common Buckwheat (Fagopyrum esculentum)
Source: Int J Mol Sci. 2019 Sep 3;20(17):4303. doi: 10.3390/ijms20174303 (PMC6747174; doi:10.3390/ijms20174303)
Supplement: Supplementary file 1 [file ijms-20-04303-s001.zip › Supplementary-proofreading/Table S1.docx]

**Table S1.** Pearson’s rank correlation of nine libraries.

| **Sample** | **S1-1** | **S1-2** | **S1-3** | **S2-1** | **S2-2** | **S2-3** | **S3-1** | **S3-2** | **S3-3** |
| --- | --- | --- | --- | --- | --- | --- | --- | --- | --- |
| **S1-1** | 1 | 0.979 | 0.985 | 0.518 | 0.466 | 0.471 | 0.246 | 0.156 | 0.193 |
| **S1-2** | 0.979 | 1 | 0.972 | 0.567 | 0.504 | 0.405 | 0.188 | 0.161 | 0.172 |
| **S1-3** | 0.985 | 0.972 | 1 | 0.483 | 0.408 | 0.397 | 0.158 | 0.133 | 0.141 |
| **S2-1** | 0.518 | 0.567 | 0.483 | 1 | 0.952 | 0.964 | 0.798 | 0.734 | 0.759 |
| **S2-2** | 0.466 | 0.504 | 0.408 | 0.952 | 1 | 0.968 | 0.776 | 0.786 | 0.751 |
| **S2-3** | 0.471 | 0.405 | 0.397 | 0.964 | 0.968 | 1 | 0.785 | 0.763 | 0.749 |
| **S3-1** | 0.246 | 0.188 | 0.158 | 0.798 | 0.776 | 0.785 | 1 | 0.983 | 0.993 |
| **S3-2** | 0.156 | 0.161 | 0.133 | 0.734 | 0.786 | 0.763 | 0.983 | 1 | 0.998 |
| **S3-3** | 0.193 | 0.172 | 0.141 | 0.759 | 0.751 | 0.749 | 0.993 | 0.998 | 1 |
