# Supplementary material for: Transcriptome Analysis Reveals Key Seed-Development Genes in Common Buckwheat (Fagopyrum esculentum)
Source: Int J Mol Sci. 2019 Sep 3;20(17):4303. doi: 10.3390/ijms20174303 (PMC6747174; doi:10.3390/ijms20174303)
Supplement: Supplementary file 1 [file ijms-20-04303-s001.zip › Supplementary-proofreading/Table S7.docx]

**Table S7.** DEGs involved in seed size.

| **Homologous** | **Gene ID** | **S1 vs S2** | | | **S2 vs S3** | | | **S1 vs S3** | | |
| --- | --- | --- | --- | --- | --- | --- | --- | --- | --- | --- |
|  |  | **FDR** | **Log2FC** | **up/down** | **FDR** | **Log2FC** | **up/down** | **FDR** | **Log2FC** | **up/down** |
| *AtIKU2* | Fes_sc0000006.1.g000111.aua.1 | 2.30E-10 | 1.76 | up | - | - | - | 0.044752 | 2.17 | up |
| *AtIKU2* | Fes_sc0002085.1.g000015.aua.1 | - | - | - | 1.13E-09- | -1.93 | down | 2.01E-08 | -1.78 | down |
| *AtAP2* | Fes_sc0008871.1.g000002.aua.1 | 2.44E-12 | -2.60 | down | - | - | - | 7.65E-07 | -1.32 | down |
| *OsGRF4* | Fes_sc0005877.1.g000008.aua.1 | 9.61E-05 | 2.04 | up | 1.56E-12 | 1.04 | up | 1.09E-08 | 3.12 | up |
| *AtDA1* | Fes_sc0000007.1.g000121.aua.1 | 2.11E-12 | -2.46 | down | - | - | - | 2.45E-05 | -1.58 | down |
| *OsCYP78A13* | Fes_sc0004649.1.g000002.aua.1 | 1.88E-05 | 1.11 | up | 3.20E-12- | -2.30 | down | 0.033268 | -1.15 | down |
| *OsSRS5* | Fes_sc0009631.1.g000002.aua.1 | 8.55E-07 | -1.12 | down | - | - | - | 5.25E-05 | -1.39 | down |
| *OsSRS5* | Fes_sc0013744.1.g000002.aua.1 | 1.47E-07 | -1.19 | down | 0.000380 | -1.52 | down | 3.37E-11 | -2.68 | down |
| *AtDWF4* | Fes_sc0000081.1.g000014.aua.1 | 0.000486 | 1.27 | up | - | - | - | - | - | - |
| *AtDET2* | Fes_sc0188737.1.g000001.aua.1 | 5.84E-07 | 1.09 | up | - | - | - | 0.001335 | 1.11 | up |
| *OsWRKY53* | Fes_sc0000044.1.g000018.aua.1 | 1.29E-07 | 1.04 | up | - | - |  | 1.50E-08 | 1.86 | up |
| *OsGIF1* | Fes_sc0000367.1.g000023.aua.1 | - | - | - | 0.017010 | 1.04 | up | 0.004865 | 1.36 | up |
| *OsSLG* | Fes_sc0013986.1.g000005.aua.1 | - | - | - | 1.16E-06 | -1.58 | down | 1.01E-08 | -1.83 | down |
| *OsCYP724B1* | Fes_sc0000554.1.g000005.aua.1 | 0.000486 | 1.27 | up | - | - | - | - | - | - |
| *AtTTG2* | Fes_sc0000097.1.g000023.aua.1 | 2.96E-04 | 2.30 | up | - | - | - | 6.61E-09 | 2.92 | up |
| *AtANT* | Fes_sc0018910.1.g000001.aua.1 | 0.001682 | 1.34 | up | 4.47E-14 | -2.89 | down | 1.36E-05 | -1.53 | down |
| *AtEOD1* | Fes_sc0001127.1.g000009.aua.1 | 0.006185 | -1.93 | down | - | - | - | - | - | - |
