# Supplementary material for: Transcriptome Analysis Reveals Key Seed-Development Genes in Common Buckwheat (Fagopyrum esculentum)
Source: Int J Mol Sci. 2019 Sep 3;20(17):4303. doi: 10.3390/ijms20174303 (PMC6747174; doi:10.3390/ijms20174303)
Supplement: Supplementary file 1 [file ijms-20-04303-s001.zip › Supplementary-proofreading/Table S8.docx]

**Table S8.** List of genes related to starch biosynthesis in common buckwheat.

| **Gene** | **Gene ID** | **Arabidopsis homologous** |
| --- | --- | --- |
| SUS | Fes_sc0000045.1.g000030.aua.1 | AT3G43190.1 |
|  | Fes_sc0000621.1.g000004.aua.1 | AT1G73370.1 |
|  | Fes_sc0001405.1.g000001.aua.1 | AT5G20830.2 |
|  | Fes_sc0002952.1.g000003.aua.1 | AT3G43190.1 |
|  | Fes_sc0003515.1.g000010.aua.1 | AT5G37180.1 |
|  | Fes_sc0006080.1.g000001.aua.1 | AT4G02280.1 |
|  | Fes_sc0006117.1.g000007.aua.1 | AT1G73370.1 |
|  | Fes_sc0007558.1.g000005.aua.1 | AT4G02280.1 |
|  | Fes_sc0023486.1.g000001.aua.1 | AT4G02280.1 |
|  | Fes_sc0028227.1.g000001.aua.1 | AT1G73370.1 |
|  | Fes_sc0030170.1.g000001.aua.1 | AT3G43190.1 \| |
|  | Fes_sc0052588.1.g000001.aua.1 | AT5G49190.1 |
|  | Fes_sc0053143.1.g000001.aua.1 | AT4G02280.1 |
|  | Fes_sc0058621.1.g000001.aua.1 | AT5G20830.2 |
|  | Fes_sc0086881.1.g000001.aua.1 | AT4G02280.1 |
|  | Fes_sc0115341.1.g000001.aua.1 | AT5G37180.1 |
|  | Fes_sc0140861.1.g000001.aua.1 | AT1G73370.1 |
|  | Fes_sc0142039.1.g000001.aua.1 | AT4G02280.1 |
|  | Fes_sc0142668.1.g000001.aua.1 | AT1G73370.1 |
|  | Fes_sc0247613.1.g000001.aua.1 | AT3G43190.1 |
|  | Fes_sc0248879.1.g000001.aua.1 | AT3G43190.1 |
|  | Fes_sc0293886.1.g000001.aua.1 | AT3G43190.1 |
|  | Fes_sc0327288.1.g000001.aua.1 | AT5G37180.1 |
|  | Fes_sc0333276.1.g000001.aua.1 | AT1G73370.1 |
|  | Fes_sc0388210.1.g000001.aua.1 | AT5G49190.1 |
| UGPase | Fes_sc0002411.1.g000014.aua.1 | AT5G17310.2 |
|  | Fes_sc0003773.1.g000005.aua.1 | AT3G56040.1 |
|  | Fes_sc0005131.1.g000007.aua.1 | AT5G17310.2 |
| AGPase | Fes_sc0000048.1.g000020.aua.1 | AT1G27680.1 |
|  | Fes_sc0000081.1.g000017.aua.1 | AT5G48300.1 |
|  | Fes_sc0000388.1.g000020.aua.1 | AT1G74910.2 |
|  | Fes_sc0006664.1.g000002.aua.1 | AT1G05610.1 |
|  | Fes_sc0012290.1.g000004.aua.1 | AT5G19220.1 |
|  | Fes_sc0015426.1.g000003.aua.1 | AT1G74910.2 |
|  | Fes_sc0034411.1.g000001.aua.1 | AT5G48300.1 |
|  | Fes_sc0045323.1.g000001.aua.1 | AT5G48300.1 |
|  | Fes_sc0051732.1.g000001.aua.1 | AT1G74910.2 |
|  | Fes_sc0073054.1.g000001.aua.1 | AT5G19220.1 |
|  | Fes_sc0131145.1.g000001.aua.1 | AT1G74910.2 |
|  | Fes_sc0167260.1.g000001.aua.1 | AT1G74910.2 |
|  | Fes_sc0232932.1.g000001.aua.1 | AT5G48300.1 |
|  | Fes_sc0233354.1.g000001.aua.1 | AT5G48300.1 |
| GBSS | Fes_sc0004292.1.g000004.aua.1 | AT1G32900.1 |
|  | Fes_sc0073997.1.g000001.aua.1 | AT1G32900.1 |
|  | Fes_sc0078760.1.g000001.aua.1 | AT1G32900.1 |
|  | Fes_sc0098294.1.g000001.aua.1 | AT1G32900.1 |
|  | Fes_sc0098658.1.g000001.aua.1 | AT1G32900.1 |
|  | Fes_sc0133745.1.g000001.aua.1 | AT1G32900.1 |
|  | Fes_sc0271790.1.g000001.aua.1 | AT1G32900.1 |
|  | Fes_sc0386817.1.g000001.aua.1 | AT1G32900.1 |
| SS | Fes_sc0000004.1.g000005.auf.1 | AT1G11720.1 |
|  | Fes_sc0000009.1.g000056.aua.1 | AT3G01180.1 |
|  | Fes_sc0000323.1.g000009.aua.1 | AT4G18240.1 |
|  | Fes_sc0002320.1.g000001.aua.1 | AT3G01180.1 |
|  | Fes_sc0006379.1.g000006.aua.1 | AT4G18240.1 |
|  | Fes_sc0002932.1.g000007.aua.1 | AT1G11720.2 |
|  | Fes_sc0003053.1.g000007.aua.1 | AT4G18240.1 |
|  | Fes_sc0007201.1.g000005.aua.1 | AT4G18240.1 |
|  | Fes_sc0009001.1.g000003.aua.1 | AT3G01180.1 |
|  | Fes_sc0010153.1.g000004.aua.1 | AT3G01180.1 |
|  | Fes_sc0014975.1.g000001.aua.1 | AT4G18240.1 |
|  | Fes_sc0016687.1.g000004.aua.1 | AT3G01180.1 |
|  | Fes_sc0022979.1.g000002.aua.1 | AT1G11720.2 |
|  | Fes_sc0023853.1.g000001.aua.1 | AT1G11720.2 |
|  | Fes_sc0043189.1.g000001.aua.1 | AT1G11720.2 |
|  | Fes_sc0047546.1.g000001.aua.1 | AT1G11720.2 |
|  | Fes_sc0057099.1.g000001.aua.1 | AT1G11720.2 |
|  | Fes_sc0069832.1.g000001.aua.1 | AT1G11720.2 |
|  | Fes_sc0076921.1.g000001.aua.1 | AT1G11720.2 |
|  | Fes_sc0080173.1.g000001.aua.1 | AT4G18240.1 |
|  | Fes_sc0088523.1.g000001.aua.1 | AT3G01180.1 |
|  | Fes_sc0094105.1.g000001.aua.1 | AT4G18240.1 |
|  | Fes_sc0099955.1.g000001.aua.1 | AT1G11720.2 |
|  | Fes_sc0127949.1.g000001.aua.1 | AT4G18240.1 |
| SBE | Fes_sc0000127.1.g000022.aua.1 | AT5G03650.1 |
|  | Fes_sc0001814.1.g000004.ana.1 | AT5G03650.1 |
|  | Fes_sc0003492.1.g000005.aua.1 | AT5G03650.1 |
|  | Fes_sc0007590.1.g000001.aua.1 | AT5G03650.1 |
| DBE | Fes_sc0000287.1.g000005.aua.1 | AT4G31770.1 |
|  | Fes_sc0000335.1.g000007.aua.1 | AT1G03310.1 |
|  | Fes_sc0001905.1.g000005.aua.1 | AT1G03310.1 |
