# Supplementary material for: Transcriptome Analysis Reveals Key Seed-Development Genes in Common Buckwheat (Fagopyrum esculentum)
Source: Int J Mol Sci. 2019 Sep 3;20(17):4303. doi: 10.3390/ijms20174303 (PMC6747174; doi:10.3390/ijms20174303)
Supplement: Supplementary file 1 [file ijms-20-04303-s001.zip › Supplementary-proofreading/Table S9.docx]

**Table S9.** Primers of sequences for qRT-PCR analysis.

| **Gene ID** | **Product Length (bp)** | **Forward primer (5`- 3`)** | **Reverse primer (5`- 3`)** |
| --- | --- | --- | --- |
| Fes_sc0060020.1.g000001.aua.1 (*FeCML*) | 156 | GATACTTGAGCTTGGAGGAC | GACTCAGCATCCTCTTCAAG |
| Fes_sc0011071.1.g000006.aua.1 (*FeCBL*) | 190 | TCTTGGTCCTGGTATGTCTG | CAGCTTATCAGCACGGACAT |
| Fes_sc0000035.1.g000053.aua.1 (*FeCDPK*) | 154 | TACGGAGAGTTTATCGCTGC | CGTCGTCTATTCCAAACTCC |
| Fes_sc0005307.1.g000002.aua.1 (*FePIF3*) | 184 | GCAAGTAGACCTTATGTGCC | GAACACAAGGCATTGGCATC |
| Fes_sc0011976.1.g000004.aua.1 (*FePY*L) | 178 | CACAACACTTCACCAGATCG | ACCAATGCTTCCTCCTTCAC |
| Fes_sc0000024.1.g000035.aua.1 (*FeABI5*) | 149 | ACACCACGGATTTGGAAGAC | CCTTAGGCATCTTCTCTTGG |
| Fes_sc0032547.1.g000002.aua.1 (*FeARF*) | 122 | CTGCTCTTTCAACGTCCTAG | GCTCAGCGTGTATGTTCTTC |
| Fes_sc0009187.1.g000001.aua.1 (*FeBRI1*) | 108 | GACATCTTACACGACCGGAA | GCAGTTGTGATGCAGGAATG |
| Fes_sc0000006.1.g000111.aua.1 (*FeIKU2*) | 138 | GGCAAGGAATCAACCCATGT | GCTTCTTACCCGTCACCAGT |
| Fes_sc0005877.1.g000008.aua.1 (*FeGRF4*) | 194 | AAAATCCATCCCAAAGCGGC | TCTTCGCTGCTATCCTCGGT |
| Fes_sc0018910.1.g000001.aua.1  (*FeANT*) | 258 | CAGCAACATCAGAATGGTGG | CTGAGAAGCCGAAATCCATG |
| Fes_sc0023486.1.g000001.aua.1 (*FeSUS*) | 122 | ATGCCTGGGTTGTATCGAGT | GTGAGTCGTTTCTCCTTCTC |
| Fes_sc0000081.1.g000017.aua.1 (*FeAGPase*) | 192 | TGATTGGAGAAGGCTGTGTC | GAGAGTTCTTACCGATACCG |
| Fes_sc0002521.1.g000007.aua.1 (*FeGBSS*) | 190 | CAGCATTCAACCCATCCATG | TTGCCCATCTGAAACCCTGT |
| Fes_sc0005785.1.g000003.aua.1 (*FeSS*) | 133 | GGAGGACTTAGGGATACTGT | ATGTTCTCACCGCATTCTCG |
| Fes_sc0000626.1.g000008.aua.1 (*FeActin7*) | 132 | CCTTGCTTCTCTTAGCACCT | TCACGACCATCTTCATCCAC |
