# Supplementary figures and images for: Transcriptome Analysis Reveals Key Seed-Development Genes in Common Buckwheat (Fagopyrum esculentum)
Source: Int J Mol Sci. 2019 Sep 3;20(17):4303. doi: 10.3390/ijms20174303 (PMC6747174; doi:10.3390/ijms20174303)

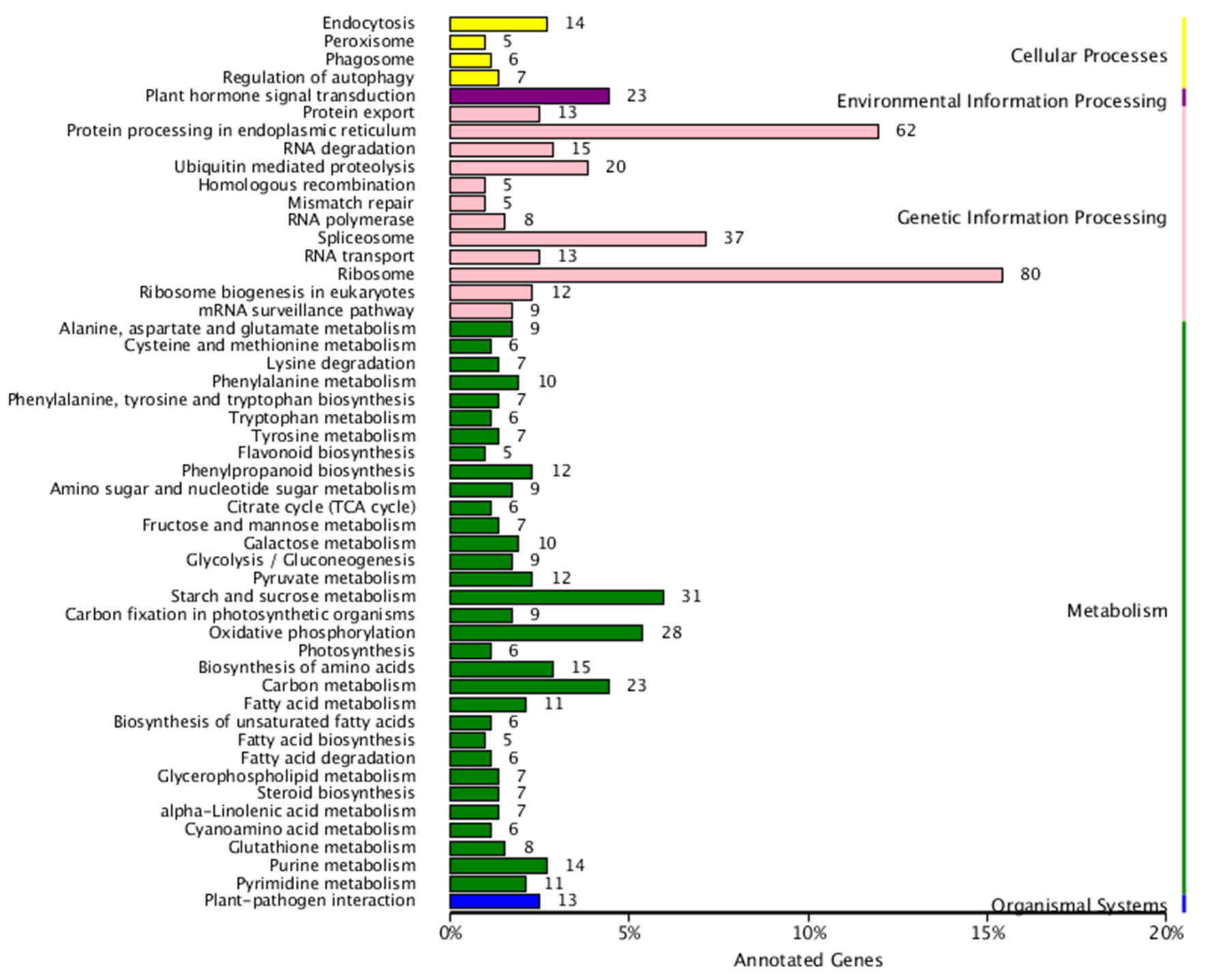

Supplement: Supplementary file 1 [file ijms-20-04303-s001.zip › Supplementary-proofreading/Figure S1.tif]

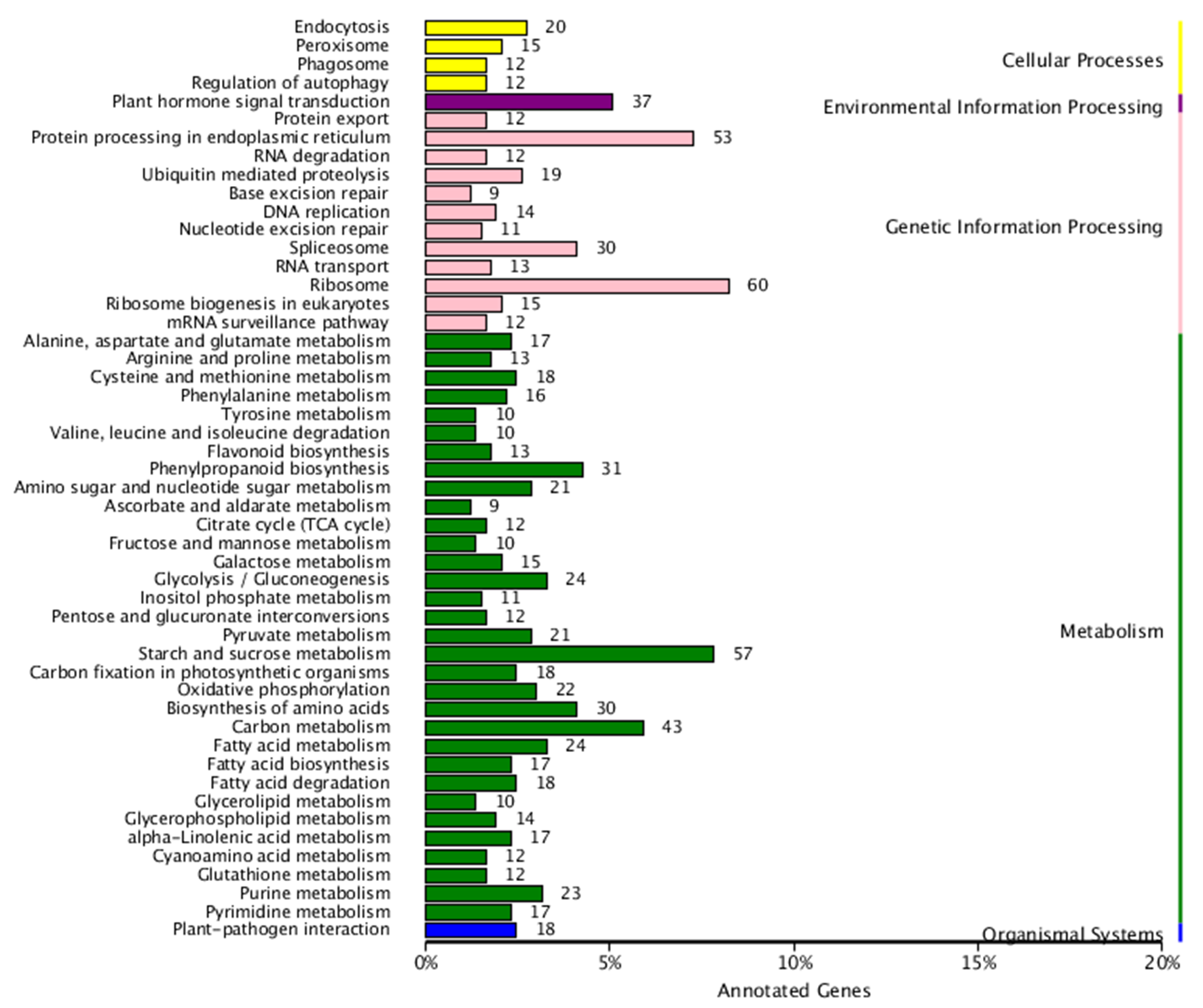

Supplement: Supplementary file 1 [file ijms-20-04303-s001.zip › Supplementary-proofreading/Figure S2.tif]
